# The association of Health-Related Quality of Life and 1-year-survival in sarcoma patients—results of a Nationwide Observational Study (PROSa)

**DOI:** 10.1038/s41416-022-01702-z

**Published:** 2022-01-20

**Authors:** Martin Eichler, Susanne Singer, Leopold Hentschel, Stephan Richter, Peter Hohenberger, Bernd Kasper, Dimosthenis Andreou, Daniel Pink, Jens Jakob, Robert Grützmann, Stephen Fung, Eva Wardelmann, Karin Arndt, Vitali Heidt, Sergio Armando Zapata Bonilla, Verena I. Gaidzik, Helena K. Jambor, Jürgen Weitz, Klaus-Dieter Schaser, Martin Bornhäuser, Jochen Schmitt, Markus K. Schuler

**Affiliations:** 1 Clinic and Polyclinic for Internal Medicine I, University Hospital Carl Gustav Carus, TU Dresden, Germany; 2 National Center for Tumor Diseases (NCT/UCC), Dresden, Germany; 3 Institute for Medical Biostatistics, Epidemiology and Informatic, University Hospital Mainz, Mainz, Germany; 4 Division of Surgical Oncology & Thoracic Surgery, Mannheim University Medical Center, University of Heidelberg, Mannheim, Germany; 5 Sarcoma Unit, Mannheim Cancer Center, Mannheim University Medical Center, University of Heidelberg, Mannheim, Germany; 6 Department of General Orthopedics and Tumor Orthopedics, University Hospital Münster, Münster, Germany; 7 Sarcoma Center Berlin-Brandenburg, Helios Hospital Bad Saarow, Bad Saarow, Germany; 8 Department of Internal Medicine C, University Hospital Greifswald, Greifswald, Germany; 9 Clinic for general, visceral, and pediatric surgery, University Hospital Goettingen, Göttingen, Germany; 10 Clinic for surgery, University Hospital Erlangen, Erlangen, Germany; 11 Clinic for general, visceral, and pediatric surgery, University Hospital Dusseldorf, Dusseldorf, Germany; 12 Gerhard-Domagk-Institute of Pathology, University Hospital Münster, Münster, Germany; 13 German Sarcoma Foundation, Woelfersheim, Germany; 14 The Scientific Institute of Office-based Hematologists and Oncologists, Cologne, Germany; 15 Clinic and Polyclinic for Internal Medicine III/ University-Centre for Tumor Diseases, University Hospital Mainz, Mainz, Germany; 16 Clinic for Internal Medicine III, University Hospital Ulm, Ulm, Germany; 17 Department of Visceral, Thoracic and Vascular Surgery, University Hospital Carl Gustav Carus, TU Dresden, Germany; 18 University Center for Orthopedics and Trauma Surgery, TU Dresden, Germany; 19 Center for Evidence-based Healthcare, University Hospital Carl Gustav Carus, Technical University Dresden, Dresden, Germany

**Keywords:** Sarcoma, Bone cancer

## Abstract

**Background:**

Sarcomas are rare cancers of high heterogeneity. Health-Related Quality of Life (HRQoL) has been shown to be a prognostic factor for survival in other cancer entities but it is unclear whether this applies to sarcoma patients.

**Patients and methods:**

HRQoL was prospectively assessed in adult sarcoma patients from 2017 to 2020 in 39 German recruiting sites using the European Organisation for Research and Treatment of Cancer Quality of Life Questionnaire (EORTC QLQ-C30). Vital status was ascertained over the course of 1 year. HRQoL domains were analysed by multivariable cox-regressions including clinical and socio-economic risk factors.

**Results:**

Of 1102 patients, 126 (11.4%) died during follow-up. The hazard ratio (HR) for global health was 0.73 per 10-point increase (95% confidence interval (CI) 0.64–0.85). HR for the HRQoL-summary score was 0.74 (CI 0.64–0.85) and for physical functioning 0.82 (CI 0.74–0.89). There was also evidence that fatigue (HR 1.17, CI 1.10–1.25), appetite loss (HR 1.15, CI 1.09–1.21) and pain (HR 1.14, CI 1.08–1.20) are prognostic factors for survival.

**Conclusion:**

Our study adds sarcoma-specific evidence to the existing data about cancer survival in general. Clinicians and care-givers should be aware of the relations between HRQoL and survival probability and include HRQoL in routine assessment.

## Introduction

Sarcomas are rare cancers with about 7000 reported new cases per year in Germany [1] and an incidence of around 5 per 100 000 per year in Europe [2]. These tumours are heterogeneous and can be grouped into many histological subtypes [3], which appear almost everywhere in the body. Sarcoma therapy is based on complex and divergent treatment algorithms [4] and relative survival of patients varies. The European rare cancer project published the relative 1- and 5-year survival rates for the years 2000–2002 showing that after 1 year 75% of soft tissue sarcoma patients, 84% of bone sarcoma and 84% of GIST patients are alive. These rates dropped to 58%, 62% and 68%, respectively, after 5 years [2]. There was a great variation in the 5-year survival rates of different subtypes, ranging from 94% in skin sarcomas to only 11% in sarcomas of the heart.

In the last decades, the investigation of Health-Related Quality of Life (HRQoL) domains gained attention as potential prognostic factors for survival in oncology. A recent systematic review and meta-analysis investigated patient-reported outcomes (PRO) across 138 studies published between 2013 and 2018 that included around 160,000 cancer patients in total [5]. In 120 of these studies, at least one PRO was reported to be prognostic for overall survival. The European Organisation for Research and Treatment of Cancer Quality of Life Questionnaire (EORTC QLQ-C30) was most often used for PRO measurement. The physical functioning scale of the EORTC QLQ-C30 was the most frequently reported independent prognostic PRO with a pooled hazard ratio of 0.88 per 10-point increase, while appetite loss and fatigue ranked second and third. Similarly, a population-based study from 2020 of 7000 patients reported that the EORTC QLQ-C30 summary score (compared to global health and physical functioning) had highest predictability of survival (0.77 per 10-point increase) [6] in 12 cancer sites combined. However, this study also reported that the analysed PROs were not of prognostic value for every investigated cancer site. Lastly, a meta-analysis of phase-II or -III randomised controlled trials showed that in 41 of 44 studies at least one HRQoL domain was significantly associated with overall survival [7]. The most commonly evaluated factors were physical functioning, global health and pain; caution is, however, necessary, as this meta-analysis also noted a lack of methodological standards in the reporting of results.

Two larger studies of HRQoL of sarcoma patients were published in 2020 [8, 9]; however, data on the relationship between HRQoL and survival in adult sarcoma patients were scarce. Another study has investigated HRQoL in pediatric osteosarcoma patients [10] and several studies have evaluated patients with advanced cancers and included smaller groups of sarcoma patients populations that were not evaluated separately [11–14]. Given that there is considerable evidence for the association between HRQoL domains and survival in several cancers, we here investigated the following open questions for sarcoma patients:

1. We hypothesised that global health, summary score, physical functioning, appetite loss, fatigue and pain are significantly associated with overall survival in sarcoma patients.
2. As there is no data yet on the relation between several other HRQoL domains assessed by the EORTC QLQ-C30 and survival, we additionally explored the relation of other available HRQoL domains and survival.
3. In the absence of established standards, we used different measurement levels of the prognostic factors studied: PROs were analysed as continuous variables, as quartiles, and in dichotomised form (thresholds).

## Patients and methods

The prospective PROSa-cohort study (Burden and Medical Care of Sarcoma in Germany: Nationwide Cohort Study Focusing on Modifiable Determinants of Patient-Reported Outcome Measures in Sarcoma Patients) (www.uniklinikum-dresden.de/prosastudie) was conducted nationwide from 09/2017 to 05/2020 in 39 study centers in Germany (NCT03521531; ClinicalTrials.gov). PROSa gathered information on a range of patient-reported outcomes (for example, HRQoL and distress) at baseline, as well as after 6 (t1) and after 12 months (t2), clinical data (such as diagnosis and treatment), as well as structural data of the participating study centers (for example, certifications and numbers of treated patients). Patients who were mentally or linguistically unable to complete questionnaires were excluded. For the present analysis, data of adult patients with histologically proven sarcoma of any entity were analysed. We analysed only participants with HRQoL data at baseline and information on survival.

Eligible patients were asked to participate at the referral centers during visits and sometimes by phone or letter. Participation required informed consent. The study was approved by the local ethics committees of the Technical University of Dresden (EK1790422017) and of the participating centers. HRQoL-data and sociodemographic data were sent by the participants to the study center by mail or online. Clinical information was submitted online by the participating centers using documentation forms. Data collection was performed using REDCap [15]. More detailed information on study design and participation have previously been published [8, 16].

### Measurement of Health-Related Quality of Life and follow-up

HRQoL was measured at baseline according to the EORTC QLQ-C30 [17]. This instrument measures in units from 0 to 100 global health/ QoL as well as 5 functioning and 9 symptom domains, where high values indicate better HRQoL (functioning domains) and higher symptom burden (symptom domains), respectively. The functioning and the symptom domains were aggregated in the EORTC QLQ-C30 summary score. [18] Potentially confounding clinical and sociodemographic variables were measured at baseline (see below).

Patients were followed up after study inclusion for 1 year. If no data on vital status was available at t2 (follow-up time), we considered them as lost to follow-up and censored them at the time of the last available data point.

### Statistics

Number of events and censoring are presented in Table 1. Continuous model variables were evaluated by mean and standard deviation (SD) if normally distributed and by median and interquartile range (IQR) if not. Categorical variables were presented with absolute and relative frequencies. All variables were stratified by vital status at last presentation (Table 2).

**Table 1:** Study population and censoring.

Population	Reduction	*N*
Participants	—	1309
No HRQoL data	197	1112
No Follow-up data	10	1102
*Analysed population*		1102
Deceased until t2	126	976
Loss to follow-up until t2/ censored before t2	28	948
Censored at t2	948	—

**Table 2:** Description study population stratified by vital status at t2.

Variable	Value	Alive at time of censoring *N* = 976 (88.6%) *N* (row%)/ mean (SD)	Deceased at t2 *N* = 126 (11.4%) *N* (row%)/ mean (SD)	All responder *N* = 1102 *N* (column%)/ mean (SD)
Sex*	Female	489 (91.1%)	48 (8.9%)	537 (48.7%)
	Male	487 (86.2%)	78 (13.8%)	565 (51.3%)
Age at baseline		56.4 (15.9)	58.2 (16.1)	56.2 (15.9)
Employment status at baseline*	Employed/ self employed	443 (91.3%)	42 (8.7%)	485 (44%)
	Unemployed	40 (87%)	6 (13%)	46 (4.2%)
	Disability pension	109 (79.6%)	28 (20.4%)	137 (12.4%)
	Early retirement/ retirement pension/ partial retirement	334 (88.1%)	45 (11.9%)	379 (34.4%)
	Housewife/ houseman	25 (96.2%)	1 (3.8%)	26 (2.4%)
	School/ apprenticeship/ study	15 (88.2%)	2 (11.8%)	17 (1.5%)
	Unknown	10 (83.3%)	2 (16.7%)	12 (1.1%)
Education (school)	None to secondary school (8/ 9 years)	229 (86.7%)	35 (13.3%)	264 (24%)
	Secondary school (10 years)	329 (87.7%)	46 (12.3%)	375 (34%)
	Vocational baccalaureate	112 (94.9%)	6 (5.1%)	118 (10.7%)
	High school/ baccalaureate	281 (88.6%)	36 (11.4%)	317 (28.8%)
	Something else/ unknown	25 (89.3%)	3 (10.7%)	28 (2.5%)
Sarcoma type—general^a^	Soft tissue sarcoma	674 /87.0)	101 (13.0)	775 (70,3)
	Bone sarcoma	178 (90.4)	19 (9.6)	197 (17.9)
	GIST	124 (12.7)	6 (4.8)	130 (11.8)
Sarcoma type***	Unclassified sarcoma	136 (83.4%)	27 (16.6%)	163 (14.8%)
	Fibroblastic, myofibroblastic, fibrohistiocytic	119 (93%)	9 (7%)	128 (11.6%)
	GIST	124 (95.4%)	6 (4.6%)	130 (11.8%)
	Liposarcoma	192 (91.9%)	17 (8.1%)	209 (19%)
	Leiomyosarcoma	117 (88.6%)	15 (11.4%)	132 (12%)
	Osteosarcoma	64 (90.1%)	7 (9.9%)	71 (6.4%)
	Synovialsarcoma	39 (81.3%)	9 (18.8%)	48 (4.4%)
	Ewing sarcoma	42 (93.3%)	3 (6.7%)	45 (4.1%)
	Chondrosarcoma	55 (85.9%)	9 (14.1%)	64 (5.8%)
	Others	88 (78.6%)	24 (21.4%)	112 (10.2%)
Site*	Abdomen/ retroperitoneum	270 (90.3%)	29 (9.7%)	299 (27.1%)
	Thorax	75 (83.3%)	15 (16.7%)	90 (8.2%)
	Pelvis	105 (85.4%)	18 (14.6%)	123 (11.2%)
	Lower limbs	365 (91.3%)	35 (8.8%)	400 (36.3%)
	Upper limbs	72 (85.7%)	12 (14.3%)	84 (7.6%)
	Head and neck	26 (74.3%)	9 (25.7%)	35 (3.2%)
	Spine (bone spine and pelvis)	38 (88.4%)	5 (11.6%)	43 (3.9%)
	Unknown/other	25 (89.3%)	3 (10.7%)	28 (2.5%)
T-stage at diagnosis	Small T1	158 (91.9%)	14 (8.1%)	172 (15.6%)
	Large (T2–T4)	459 (89.6%)	53 (10.4%)	512 (46.5%)
	Other/ unknown	359 (85.9%)	59 (14.1%)	418 (37.9%)
Grading at diagnosis*	Low grade	129 (95.6%)	6 (4.4%)	135 (12.3%)
	High grade	524 (87.2%)	77 (12.8%)	601 (54.5%)
	Not applicable/ unknown	323 (88.3%)	43 (11.7%)	366 (33.2%)
Time since diagnosis (baseline)**	0–<0.5 year	178 (84.8%)	32 (15.2%)	210 (19.1%)
	0.5–<1 year	100 (81.3%)	23 (18.7%)	123 (11.2%)
	1–<2 years	145 (87.9%)	20 (12.1%)	165 (15%)
	2–<5 years	269 (91.8%)	24 (8.2%)	293 (26.6%)
	More than 5 years	284 (91.3%)	27 (8.7%)	311 (28.2%)
Tumour recurrence until baseline**	No recurrence	719 (90.4%)	76 (9.6%)	795 (72.1%)
	Recurrence	234 (83.9%)	45 (16.1%)	279 (25.3%)
	Suspicion/ unknown	23 (82.1%)	5 (17.9%)	28 (2.5%)
Metastasis until baseline***	No metastasis	579 (96.2%)	23 (3.8%)	602 (54.6%)
	Metastasis	253 (72.5%)	96 (27.5%)	349 (31.7%)
	Unknown/ suspicion	144 (95.4%)	7 (4.6%)	151 (13.7%)
Disease status at baseline***	Complete remission	482 (98.6%)	7 (1.4%)	489 (44.4%)
	Partial remission + stable disease	291 (88.4%)	38 (11.6%)	329 (29.9%)
	Tumour progress	103 (64.4%)	57 (35.6%)	160 (14.5%)
	Unknown or not accessible	100 (80.6%)	24 (19.4%)	124 (11.3%)
Comorbidities**	None	492 (91.1%)	48 (8.9%)	540 (49%)
	1	308 (85.6%)	52 (14.4%)	360 (32.7%)
	2	135 (88.8%)	17 (11.2%)	152 (13.8%)
	3	35 (89.7%)	4 (10.3%)	39 (3.5%)
	4 and more	6 (54.5%)	5 (45.5%)	11 (1%)
Surgery until baseline***	No	100 (75.2%)	33 (24.8%)	133 (12.1%)
	Yes	876 (90.4%)	93 (9.6%)	969 (87.9%)
Chemotherapy until baseline***	No	556 (95.9%)	24 (4.1%)	580 (52.6%)
	Yes	420 (80.5%)	102 (19.5%)	522 (47.4%)
Radiotherapy until baseline	No	601 (89.3%)	72 (10.7%)	673 (61.1%)
	Yes	375 (87.4%)	54 (12.6%)	429 (38.9%)
Treatment intention***/^a^	Curative	783 (95.5%)	37 (4.5%)	820 (74.4%)
	Palliative	175 (67.3%)	85 (32.7%)	260 (23.6%)
	Unknown	18 (81.8%)	4 (18.2%)	22 (2%)

*N* = 1102.

*p < 0.05.

**p  < 0.01.

***p < 0.001 (chi square).

^a^No model variable.

Multivariable cox-regressions were fitted to test for differences in survival between different HRQoL levels. HRQoL domains were evaluated in continuous form (model 1). Additionally we analysed them as quartiles (model 2) and in dichotomous form (model 3), using the thresholds of Giesinger et al. [19]. These indicate the proportion of patients with clinical important symptoms and limitations in the HRQoL domains. Proportional hazard assumption was tested using log minus log plots.

We considered global health, summary score, physical functioning, appetite loss, fatigue and pain as likely prognostic factors of survival. For those, results of model 2 were displayed as survival curves [20]. The other domains of the EORTC QLQ-C30 were evaluated exploratively (Table 3).

**Table 3:** Results of the mulitvariable cox-regression.

QoL domain	Univariate	Model 1	Model 2				Model 3			
	Per 10 points HR (95% CI)	Per 10 points HR (95% CI)	Quartiles [range] (%)		Deceased (%)	Model HR (95% CI)	Clinical important restrictions or symptoms [range] (%)		Deceased (%)	Model HR (95% CI)
Summary score	**0.68** (0.60–0.76)	**0.74** (0.64–0.85)	4.Qu. [>86.4–100]	271 (25.1)	10 (3.7)	ref.	n.a.	n.a.	n.a.	n.a.
			3.Qu. [>73.6–86.4]	271 (25.1)	16 (5.9)	1.01 (0.43–2.38)				
			2.Qu. [>57.8–73.6]	268 (24.8)	39 (14.6)	**2.27** (1.06–4.88)				
			1.Qu. [0–57.8]	270 (25.0)	60 (22.0)	**3.65** (1.73–7.69)				
Global health	**0.69** (0.62–0.76)	**0.73** (0.64–0.82)	4.Qu. [83.3–100]	262 (23.9)	5 (1.9)	ref.	n.a.	n.a.	n.a.	n.a.
			3.Qu. [66.7–75.0]	289 (26.3)	26 (9.0)	**3.11** (1.12–8.59)				
			2.Qu. [50.0–58.3]	264 (24.1)	34 (12.9)	**3.58** (1.31–9.78)				
			1.Qu. [0–41.7]	282 (25.7)	61 (21.6)	**8.29** (3.13–22.00)				
Physical functioning	**0.77** (0.71–0.83)	**0.82** (0.74–0.89)	4.Qu. [93.3–100]	336 (30.5)	16 (4.8)	ref.	No [86.7–100]	447 (40.6)	24 (5.4)	ref.
			3.Qu. [80.0–91.7]	231 (21.0)	18 (7.8)	1.09 (0.52–2.28)				
			2.Qu. [58.3–73.3]	248 (22.5)	32 (12.9)	**2.42** (1.23–4.78)	Yes [0–80.0]	653 (59.4)	102 (15.6)	**2.35** (1.43–3.87)
			1.Qu. [0–53.3]	285 (25.9)	60 (21.1)	**2.89** (1.52–5.51)				
Role functioning	**0.82** (0.77–0.88)	**0.91** (0.84–0.97)	4.Qu. [100]	240 (21.8)	10 (4.2)	ref.	No [66.7–100]	542 (49.3)	33 (6.1)	ref.
			3.Qu. [66.7–83.3]	302 (27.5)	23 (7.6)	0.99 (0.45–2.18)				
			2.Qu. [33.3–50.0]	315 (28.6)	44 (14.0)	1.53 (0.73–3.23)	Yes [0–50.0]	558 (50.7)	93 (16.7)	**1.67** (1.07–2.62)
			1.Qu. [0–16.7]	243 (22.1)	49 (20.2)	1.85 (0.86–3.98)				
Emotional functioning	**0.89** (0.82–0.95)	**0.89** (0.81–0.97)	4.Qu. [83.3–100]	302 (27.5)	27 (8.9)	ref.	No [75.0–100]	409 (37.2)	35 (8.6)	ref.
			3.Qu. [66.7–77.8]	239 (21.7)	16 (6.7)	0.73 (0.38–1.43)				
			2.Qu. [41.7–58.3]	331 (30.1)	43 (13.0)	1.26 (0.74–2.14)	Yes [0–66.7]	690 (62.8)	90 (13.0)	1.53 (0.98–2.37)
			1.Qu. [0–33.3]	227 (20.7)	39 (17.2)	**2.07** (1.18–3.63)				
Cognitive functioning	**0.92** (0.86–0.98)	0.95 (0.89–1.02)	4.Qu. [100]	452 (41.1)	41 (9.1)	ref.	No [83.3–100]	672 (61.0)	65 (9.7)	ref.
			3.Qu. [83.3]	220 (20.0)	24 (10.9)	1.32 (0.75–2.33)				
			2.Qu. [66.7]	205 (18.6)	28 (13.7)	**2.11** (1.22–3.62)	Yes [0–66.7]	429 (39.0)	61 (14.2)	**1.52** (1.02–2.24)
			1.Qu. [0–50.0]	224 (20.3)	33 (14.7)	1.42 (0.86–2.35)				
Social functioning	**0.80** (0.75–0.86)	**0.88** (0.81–0.94)	4.Qu. [100]	236 (21.5)	6 (2.5)	ref.	No [66.7–100]	596 (54.2)	32 (5.4)	ref.
			3.Qu. [66.7–83.3]	360 (32.7)	26 (7.2)	1.9 (0.75–4.82)				
			2.Qu. [33.3–50.0]	283 (25.7)	45 (15.9)	**2.72** (1.09–6.82)	Yes [0–50.0]	504 (45.8)	94 (18.7)	**2.07** (1.33–3.21)
			1.Qu. [0–16.7]	221 (20.1)	49 (22.2)	**4.23** (1.71–10.44)				
Fatigue	**1.22** (1.16–1.29)	**1.17** (1.10–1.25)	1.Qu. [0–16.7]	238 (21.6)	9 (3.8)	ref.	No[0–33.3]	539(49.0)	29(5.4)	ref.
			2.Qu. [22.2–33.3]	301 (27.4)	20 (6.6)	1.57 (0.68–3.64)				
			3.Qu. [44.4–55.6]	229 (20.8)	27 (11.8)	**2.34** (1.04–5.26)	Yes [44.4–100]	561 (51.0)	97 (17.3)	**2.58** (1.64–4.06)
			4.Qu. [66.7–100]	332(30.2)	70 (21.1)	**4.21** (1.98–8.94)				
Nausea vomiting	**1.17** (1.11–1.24)	**1.18** (1.10–1.27)	1.Qu. [0]	799 (72.6)	63 (7.9)	ref.	No [0]	799 (72.6)	63 (7.9)	ref.
			2.Qu. [16.7]	153 (13.9)	27 (17.6)	1.57 (0.93–2.66)				
			3.Qu. [33.3]	77 (7.0)	19 (24.7)	**2.74** (1.53–4.90)	Yes [16.7–100]	302 (27.4)	63 (20.9)	**2.17** (1.45–3.25)
			4.Qu. [50.0–100]	72 (6.5)	17 (23.6)	**3.01** (1.63–5.55)				
Pain	**1.14** (1.09–1.19)	**1.14** (1.08–1.20)	1.Qu. [0]	343 (31.2)	24 (7.0)	ref.	No [0–16.7]	490 (44.5)	36 (7.3)	ref.
			2.Qu. [16.7]	147 (13.4)	12 (8.2)	1.19 (0.56–2.56)				
			3.Qu. [33.3–50.0]	334 (30.3)	36 (10.8)	1.55 (0.87–2.74)	Yes [33.3–100]	611 (55.5)	90 (14.7)	**2.02** (1.30–3.15)
			4.Qu. [66.7–100]	277 (25.2)	54 (19.5)	**2.99** (1.72–5.19)				
Dyspnoea	**1.17** (1.12–1.23)	**1.09** (1.04–1.15)	1.Qu. [0]	554 (50.7)	38 (6.9)	ref.	No [0]	554 (50.7)	38 (6.9)	ref.
			2.Qu. [33.3]	299 (27.4)	32 (10.7)	1.22 (0.72–2.07)				
			3.Qu. [66.7]	187 (17.1)	38 (20.3)	**2.03** (1.24–3.34)	Yes [33.3–100]	539 (49.3)	87 (16.1)	**1.68** (1.10–2.57)
			4.Qu. [100]	53 (4.8)	17 (32.1)	**2.24** (1.16–4.31)				
Insomnia	1.05 (0.995–1.1)	1.04 (0.98–1.09)	1.Qu. [0]	358 (32.6)	36 (10.1)	ref.	No [0–33.3]	710 (64.7)	71 (10.1)	ref.
			2.Qu. [33.3]	352 (32.1)	36 (10.2)	0.97 (0.57–1.66)				
			3.Qu. [66.7]	245 (22.3)	32 (13.1)	1.19 (0.69–2.03)	Yes [66.7–100]	387 (35.3)	54 (14.0)	1.28 (0.86–1.90)
			4.Qu. [100]	142 (12.9)	22 (15.5)	1.41 (0.76–2.6)				
Appetite loss	**1.18** (1.13–1.23)	**1.15** (1.09–1.21)	1.Qu. [0]	722 (65.6)	49 (6.8)	ref.	No [0–33.3]	930 (84.5)	82 (8.8)	ref.
			2.Qu. [33.3]	208 (18.9)	33 (15.9)	**2.10** (1.28–3.44)				
			3.Qu. [66.7]	127 (11.5)	31 (24.4)	**3.56** (2.12–5.99)	Yes [66.7–100]	171 (15.5)	44 (25.7)	**2.68** (1.76–4.07)
			4.Qu. [100]	44 (4.0)	13 (29.5)	**3.43** (1.71–6.88)				
Constipation	**1.12** (1.07–1.17)	**1.07** (1.01–1.13)	1.Qu. [0]	768 (70.0)	66 (8.6)	ref.	No [0–33.3]	949 (86.5)	90 (9.5)	ref.
			2.Qu. [33.3]	181 (16.5)	24 (13.3)	1.12 (0.67–1.87)				
			3.Qu. [66.7]	91 (8.3)	25 (27.5)	**1.96** (1.16–3.31)	Yes [66.7–100]	148 (13.5)	36 (24.3)	**1.80** (1.16–2.79)
			4.Qu. [100]	57 (5.2)	11 (19.3)	1.66 (0.82–3.37)				
Diarrhea	**1.08** (1.03–1.14)	1.06 (0.99–1.13)	1.Qu. [0]	814 (74.1)	78 (9.6)	ref.	No[0]	814 (74.1)	78 (9.6)	ref.
			2.Qu. [33.3]	167 (15.2)	30 (18.0)	1.45 (0.89–2.36)				
			3.Qu. [66.7]	85 (7.7)	11 (12.9)	1.15 (0.56–2.36)	Yes [33.3–100]	285 (25.9)	48 (16.8)	1.44 (0.96–2.18)
			4.Qu. [100]	33 (3.0)	7 (21.2)	2.12 (0.87–5.15)				
Financial difficulties	**1.06** (1.02–1.11)	**1.07** (1.01–1.13)	1.Qu. [0]	600 (54.8)	54 (9.0)	ref.	No [0]	600 (54.8)	54 (9.0)	ref.
			2.Qu. [33.3]	236 (21.6)	29 (12.3)	**1.76** (1.04–2.98)				
			3.Qu. [66.7]	164 (14.9)	30 (18.3)	**1.99** (1.18–3.36)	Yes [33.3–100]	494 (45.2)	71 (14.4)	**1.81** (1.18–2.78)
			4.Qu. [100]	94 (8.6)	12 (12.8)	1.55 (0.72–3.3)				

Variables in the model: sex, age at baseline, employment status at baseline, school education, sarcoma type, tumour site, grading at diagnosis, tumour size at diagnosis, time since diagnosis, tumour recurrence until baseline, metastasis until baseline, disease status at baseline, comorbidities, surgery, chemotherapy, radiotherapy until baseline. Boldface = statistically significant at *p* < 0.05.

*HR* hazard ratio, *95% CI* 95% confidence intervall, *ref* reference, *n.a.* not available.

To adjust for potential confounding, we included socio-economic as well as clinical variables in the models, namely (variable values are shown in Table 2) sex, age at baseline, employment status at baseline, school education, sarcoma type, tumour site, grading at diagnosis, tumour size at diagnosis, time since diagnosis, tumour recurrence until baseline, metastasis until baseline, disease status at baseline, comorbidities at baseline and received treatments until baseline (surgery, chemotherapy, radiotherapy).

Statistical analyses were performed with SPSS V.27 (IBM Corporation, Armonk, New York, USA).

## Results

### Participation and sample description

After excluding patients without HRQoL or without follow-up data, 1102 patients could be included in the analysis (Table 1). Of those, 126 died until t2 and 28 were censored before t2. Patients were almost at gender parity with nearly half (49%) of analysed patients being female. The mean age of all patients was 56.2 years and 44% were employed at baseline. Seventy percent of analysed patients had a soft tissue sarcoma, 18% a bone sarcoma and 12% a GIST. Forty-eight percent of patients had an extremity sarcoma (Table 2). Forty-four percent of patients were in complete remission while 15% had progressive disease. Thirty-two percent were metastasised (Table 2).

We then tested if there are observable differences in survival between different HRQoL levels and evaluated HRQoL domains in continuous form (model 1), as quartiles (model 2) and in dichotomous (model 3) form.

### Model 1—HRQoL as continuous scale

In the multivariable models, global health had the biggest impact on survival with a hazard ratio (HR) of 0.73 per 10-point increase (95% confidence interval (95% CI) 0.64–0.85). The HR for the summary score was 0.74 (95% CI 0.64–0.85) and for physical functioning 0.82 (95% CI 0.74–0.89). With the exception of cognitive functioning, other functioning scales showed significant results as well (Table 3). As hypothesised, fatigue (HR 1.17, 95% CI 1.10–1.25), appetite loss (HR 1.15, 95% CI 1.09–1.21) and pain (HR 1.14, 95% CI 1.08–1.20) were significant prognostic factors for survival. Furthermore, nausea/vomiting, dyspnoea, constipation and financial difficulties reached significance among the symptom scales (Table 3).

### Model 2—HRQoL as quartiles

**Fig. 1:**
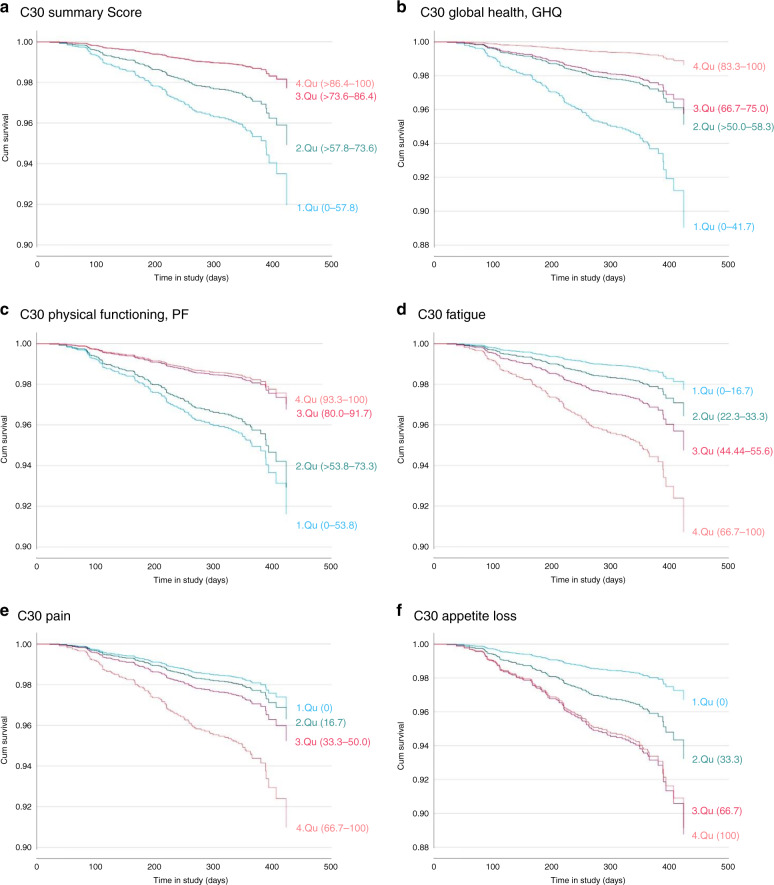
Results of the multivariable Cox-regression. Survival curves for summary score (**a**), global health (**b**), physical functioning (**c**), fatigue (**d**), pain (**e**), appetite loss (**f**). *N* = 1102. Events = 126. Variables in the model: sex, age at baseline, employment status at baseline, school education, sarcoma type, tumour site, grading at diagnosis, tumour size at diagnosis, time since diagnosis, tumour recurrence until baseline, metastasis until baseline, disease status at baseline, comorbidities, surgery, chemotherapy, radiotherapy until baseline.

When comparing the least affected quartile of patients (1. Qu) with the most affected quartile (4. Qu), we observed the highest risk of mortality associated with global health (HR 8.29, 95% CI 3.13–22.00, Fig. 1b), followed by the summary score (HR 3.65, 95% CI 1.73–7.69, Fig. 1a) and physical functioning (HR 2.89, 1.52–5.51, Fig. 1c). Further functioning scales showed significant results as well (Table 3). As hypothesised, among the symptom scales fatigue (HR 4.21, 95% CI 1.98–8.94, Fig. 1d), appetite loss (HR 3.43, 95% CI 1.71–6.88, Fig. 1f), and pain (HR 2.99, 95% CI 1.72–5.19, Fig. 1e) were significant prognostic factors for survival. Among the other symptoms scales results for nausea/ vomiting, dyspnea and constipation reached significance comparing the first with the fourth quartile. (Table 3, Fig. 1)

### Model 3—thresholds

No validated thresholds for summary score and for global health are available. Patients with clinical important restrictions in physical functioning showed a higher risk for mortality (HR 2.35, 95%-CI 1.43–3.87). Significant hazard ratios could also be observed in role, cognitive and social functioning (Table 3). As hypothesised, fatigue (HR 2.58, 95% CI 1.64–4.06), appetite loss (2.68, 95% CI 1.76–4.07) and pain (2.02, 95% CI 1.30–3.15) were significant prognostic factors for survival from the symptom scale. With the exception of insomnia and diarrhea, there was also evidence for significance in all other symptom scales. (Table 3)

## Discussion

### Results in context

Our results demonstrate that HRQoL domains are an independent prognostic factor for survival in sarcoma patients. The domains global health, physical functioning, fatigue, pain and appetite loss as well as the summary score have been previously associated with survival in cancer patients in general [5–7] and reached significance in the continuous, quartile and threshold models in our analysis. Additionally, we found significant associations between survival and the exploratively analysed HRQoL domains. Dyspnoea, nausea/ vomiting and social functioning showed significant associations in all three models, while in contrast insomnia and diarrhea were not associated with survival in any of the three models. The observed associations between dyspnoea, nausea/ vomiting and social functioning are noteworthy as they are less frequently discussed in the literature.

A key purpose of HRQoL domains in clinical settings is to measure aspects of disease burden that are not fully captured by assessing factors like disease stage, comorbidities or performance status (PS) alone. At present, the relation between PS and physical functioning, and which kind of measurement is more appropriate for different purposes, is still being discussed [21, 22]. PS und physical functioning could be considered as evaluations of the same complex status but from different perspectives and evidence suggests that some subjective self-reported toxicities may be missed by the examining physician [23]. Al-Rashdan et al. recently showed that PS und physical functioning were similarly predictive for overall survival [24]. Our analysis demonstrated that global health and the C30 summary score each had greater effect than physical functioning on predicting patient outcomes. This indicates that measuring physical functioning alone might not suffice to completely assess how disease severity is linked with survival from a patient’s perspective. The C30 summary score, which comprises all domains of the EORTC except financial problems, could alternatively provide a more comprehensive view on how survival and disease severity are linked. It is noteworthy that the generic global health domain with only two questions, on general life quality and health status, reached a similar effect size as the summary score.

At present, we are not able to answer the question if there are sarcoma-specific associations between HRQoL and survival. This is partly due to the fact that studies comparing individual cancer entities across all QoL domains with respect to survival are still lacking. Sarcomas are a highly heterogenous group of diseases with different QoL profiles [9]. An evaluation of the HRQoL of our PROSa-cohort previously revealed particular burdens in social and role functioning [8]. These may contribute to the observed association between social functioning and survival in this analysis.

In our study, continuous, quartiles and dichotomous measurement levels reached similar results. To our knowledge, there are no evaluated standards for the most appropriate form of measurement level—should a continuous scale be used or should the population be divided into groups (and if so in how many)? It is possible that no one size fits all solution exists and that domain-specific solutions have to be found. According to our results for social functioning and fatigue domains a ‘continuous’ presentation appears most appropriate due to its linear relationship; for others like global health, dyspnoea and appetite loss, we observed that a categorical approach with one or two thresholds appears useful. To give to examples: We found the most pronounced differences between the patient quartile with the best and the patient quartile with the worst global health score. With regard to the summary score, there seem to be no differences regarding survival in the least affected two quartiles of the population. Survival probabilities differed from the third quartile onward. However, as our study was not designed to evaluate thresholds but addressed the preceding question, whether there are associations between HRQoL and survival at all, it would be an overreach to derive cut-off values from these observations. In order to do that, a number of questions would need to be discussed, which are beyond the scope of this paper. For example, it should be clarified to what extent one should ask about the clinical relevance of the observed HR and whether different purposes (patient communication, medical interventions) require different thresholds.

### Strengths and limitations

To date the PROSa study is one of the largest studies on HRQoL in sarcoma patients worldwide. In this analysis, we demonstrated an association between HRQoL and survival in sarcoma patients. Patients from 39 hospitals and medical offices were included. The participating centers comprehensively represent the aspects of sarcoma treatment in Germany and have a large network of referring institutions [16].

The study potentially may be subject to a selection bias as the majority of our patients were recruited in university hospitals and/or specialised centers. Selection biases are also possible at the patient level with a possible sick survivor bias, as healthy survivors are less likely to frequent recruiting study centers over time. Another bias may be sociodemographic selection, which is a factor in any observational study. These factors, however, in our opinion will not profoundly impact or influence the group comparisons made.

The possibility of undetected systematic confounding is inherent in any observational study. We measured a broad variety of potentially confounding variables. We were not able to include performance status (PS) in our analysis as it is not routinely collected at hospital visits. We would not consider PS as a confounding variable, but to compare the effect size of the measurements, inclusion of PS would have been beneficial. We did not include time-dependent variables in our analysis. Time-dependent variables would have resulted in somewhat more precise results, but at the same time would have further restricted the time horizon of the prognosis.

## Conclusion

HRQoL domains are independent prognostic factors for survival in sarcoma patients. All hypothesised HRQoL domains, namely global health, summary score, physical functioning, fatigue, pain and appetite loss were significantly associated with survival. Our analysis therefore adds disease-specific evidences to the already existing data reported for cancer patients in general. This opens the possibility for further studies that can firmly establish the potentially domain-specific relationship between HRQoL and survival probability. It would benefit patients if clinicians and care-givers would monitor HRQoL domains in patients on a regular basis. Future studies should evaluate whether specific interventions to improve HRQoL domains might have a positive influence on patient survival.

## Data Availability

The datasets used and/or analysed during the current study are available from the corresponding author on reasonable request.
